# Identification of Key Osteoarthritis-Associated Genes Based on DNA Methylation

**DOI:** 10.3390/ijms27083388

**Published:** 2026-04-09

**Authors:** Jian Zhao, Changwu Wu, Zhejun Kuang, Han Wang, Lijuan Shi

**Affiliations:** 1 School of Computer Science and Technology, Changchun University, Changchun 130022, China; zhaojian@ccu.edu.cn (J.Z.);; 2 Jilin Provincial Key Laboratory of Human Health Status Identification Function & Enhancement, Changchun 130022, China; 3 Key Laboratory of Intelligent Rehabilitation and Barrier-Free for the Disabled, Changchun University, Ministry of Education, Changchun 130022, China; 4 School of Information Science and Technology, Institute of Computational Biology, Northeast Normal University, Changchun 130117, China; 5 School of Elite Biomedical Engineers, China Pharmaceutical University, Nanjing 210009, China; 6 Ningxia Key Laboratory of Bioimaging and Intelligent Diagnostics, Yinchuan 750021, China; 7 College of Electronic Information Engineering, Changchun University, Changchun 130022, China

**Keywords:** osteoarthritis, DNA methylation, machine learning, deep learning, feature fusion, enrichment analysis, protein–protein interaction analysis, effector genes

## Abstract

Osteoarthritis (OA) is a complex degenerative joint disease for which early diagnosis and clear molecular characterization remain limited. DNA methylation has been increasingly recognized as an important regulatory factor in OA pathogenesis. In this study, we proposed an integrative computational framework combining statistical analysis, machine learning, deep learning, and functional genomics to identify and validate OA-associated genes and methylation biomarkers for diagnostic and biological interpretation. Candidate CpG sites were obtained using two complementary strategies: differential methylation analysis and selection of loci located near transcription start sites of previously reported OA-related genes. Key features were further refined using support vector machine recursive feature elimination and random forest algorithms. Based on the selected loci, we developed a feature-fusion diagnostic model that combines Transformer and convolutional neural networks with adaptive weighting to capture both global dependency structures and local methylation patterns. A panel of 220 methylation sites demonstrated stable and reproducible diagnostic performance in an independent cohort. Functional annotation and pathway analysis highlighted several established OA-associated genes, including *TGFBR2*, *SMAD3*, *PPARG*, and *MAPK3*, and suggested *INHBB* as a potential novel effector gene, with additional support for *AMH* and *INHBE* involvement. Overall, this study presents a robust methylation-based framework for identifying key OA-associated genes and provides new insights into the epigenetic mechanisms underlying OA.

## 1. Introduction

Osteoarthritis (OA) is a degenerative joint disorder that predominantly affects middle-aged and elderly populations, emerging as one of the fastest-growing global public health burdens worldwide [1]. Epidemiological data indicate that approximately 595 million individuals are currently living with OA globally, with a striking 132% increase in prevalence between 1990 and 2020 [2]. It is projected that the global OA patient population will approach one billion by 2050 [2]. OA involves coordinated pathological changes across multiple joint tissues, including progressive articular cartilage degeneration, subchondral bone sclerosis and remodeling, synovial inflammation, osteophyte formation, and chronic pain [3,4]. Currently, clinical diagnosis of OA primarily relies on imaging assessments such as computed tomography and magnetic resonance imaging, which are typically performed after clear clinical symptoms, including pain and joint dysfunction, have developed at relatively advanced stages of the disease [5]. Despite the rising global prevalence of OA, current pharmacological treatments mainly rely on symptom-modifying osteoarthritis drugs (SMOADs), which are primarily used to alleviate pain and improve joint function. However, these therapies mainly provide symptomatic relief rather than altering disease progression, and their long-term use may lead to adverse effects and potential toxicity [6]. As an alternative therapeutic strategy, disease-modifying osteoarthritis drugs (DMOADs) have been proposed to target the underlying pathological processes of OA and potentially slow or halt disease progression. Nevertheless, the clinical development of DMOADs remains challenging due to limited efficacy and the lack of well-validated molecular targets [7].

Epigenetics refers to molecular mechanisms that regulate gene expression through reversible, heritable chemical modifications without altering the underlying DNA sequence, thereby shaping biological phenotypes [8]. Epigenetic regulation encompasses multiple processes closely linked to gene expression, including DNA methylation, histone modifications, chromatin remodeling, and regulation mediated by non-coding RNAs [8]. Among these mechanisms, DNA methylation, which predominantly occurs at cytosine residues within CpG islands, is recognized as one of the most important and extensively studied epigenetic processes [9]. Depending on the genomic context of CpG sites, their methylation status can either promote or repress gene expression [10]. Accumulating evidence further supports that aberrant DNA methylation constitutes a critical molecular underpinning of OA onset, progression, and the development of pro-inflammatory phenotypes [11].

With the rapid advancement of artificial intelligence technologies, machine learning approaches have been widely applied in the auxiliary diagnosis of various diseases [12]. Previous studies have demonstrated that traditional machine learning models, such as Support Vector Machines (SVMs), Random Forests (RF), and Extreme Gradient Boosting, can effectively perform classification and diagnosis of complex diseases, including rheumatoid arthritis and Alzheimer’s disease, with favorable predictive performance and clinical applicability [13,14]. In the context of OA research, traditional machine learning methods have also been employed in the screening of DNA methylation biomarkers. For example, one study analyzed samples from hip osteoarthritis, healthy hip, and knee osteoarthritis, successfully identifying a biomarker panel comprising 12 methylation sites, and revealed that disease-associated DNA methylation changes are more pronounced than joint-specific differences [15]. Meanwhile, the application of deep learning models in disease diagnosis has continued to expand. For instance, Convolutional Neural Network (CNN)-based approaches have been widely adopted for the automated recognition and grading of medical imaging data across a broad range of diseases, demonstrating robust performance in feature extraction and classification tasks [16]. Transformer architectures have shown strong capability in disease diagnosis from medical images by enabling effective modeling of complex spatial patterns and global contextual information [17]. In OA diagnosis, deep learning models have been used to extract features from joint imaging data, achieving high-precision assessment of disease severity and further demonstrating the advantages of deep learning in this field [18]. Nevertheless, existing approaches for early OA diagnosis remain limited, and laboratory assays continue to lack highly specific and stable biomarkers [19]. These limitations have, to a certain extent, hindered the early detection of OA and the effective evaluation of disease progression. Therefore, the identification and validation of reliable molecular biomarkers are of great significance for early OA diagnosis and dynamic disease monitoring. During OA pathogenesis, aberrant DNA methylation plays a pivotal role in shaping disease phenotypes by regulating the expression of key genes. Increased DNA methylation in human knee OA cartilage was shown to be associated with reduced expression of multiple transcription factors, including Atonal BHLH Transcription Factor 8 (*ATOH8*), MAF BZIP Transcription Factor F (*MAFF*), Nuclear Receptor Corepressor 2 (*NCOR2*), T-Box Transcription Factor 4 (*TBX4*), Zinc Finger And BTB Domain Containing 16 (*ZBTB16*), and Zinc Fingers And Homeoboxes 2 (*ZHX2*), and pharmacological demethylation partially restored their expression, indicating that epigenetic silencing of transcriptional regulators contributes to OA-related cartilage degeneration [20]. DNA methyltransferases (DNMTs) have been shown to promote A Disintegrin And Metalloproteinase With Thrombospondin Motifs 5 (*ADAMTS-5*) expression by reducing demethylation levels within its promoter region [21]. Moreover, hypermethylation of the sirtuin 1 promoter impairs the binding affinity of CCAAT/Enhancer-Binding Protein Alpha (C/EBPα), leading to reduced *SIRT1* expression, increased acetylation of Nuclear Factor Kappa-B (NF-κB) p65, and ultimately exacerbated inflammatory responses and catabolic processes in cartilage tissue [22]. Similarly, hypermethylation of the Peroxisome Proliferator Activated Receptor Gamma (*PPARG*) promoter suppresses PPARγ expression, thereby intensifying oxidative stress and inflammatory responses and accelerating OA progression [23]. Within this research framework—where DNA methylation regulates gene expression and subsequently modulates disease phenotypes—the identification of combinatorial DNA methylation biomarkers with diagnostic value represents a promising strategy for early OA detection [24].

In OA research, continuous advances in high-throughput sequencing technologies, integrative analyses across multiple omics layers, and bioinformatics methodologies have facilitated substantial progress in the systematic identification and functional validation of key OA effector genes [25]. An increasing number of potential pathogenic genes and signaling pathways have been identified and experimentally validated in vitro and in vivo, elucidating their specific roles in cartilage metabolism, inflammatory regulation, and extracellular matrix remodeling, thereby providing accumulating evidence for a deeper understanding of OA molecular pathogenesis [26]. Based on a comprehensive genome-wide association study (GWAS) involving nearly two million participants, researchers integrated multi-omics evidence—including expression quantitative trait loci (eQTL), high-throughput chromosome conformation capture (Hi-C), and DNA methylation data—to systematically identify approximately 700 effector genes with potential direct implications in the pathogenesis of OA [27]. Transcriptome-wide association studies (TWAS) implemented with the functional summary-based imputation (FUSION) algorithm enabled the identification of seven high-confidence, potentially druggable gene targets for OA, namely Glycine C-Acetyltransferase (*GCAT*), Mitogen-Activated Protein Kinase 3 (*MAPK3*), Macrophage Stimulating 1 Receptor (*MST1R*), Phosphofructokinase, Muscle (*PFKM*), RAD9 Checkpoint Clamp Component A (*RAD9A*), SMAD Family Member 3 (*SMAD3*), and Ubiquitin Specific Peptidase 8 (*USP8*) [28]. Further studies combining TWAS with chemical gene set enrichment analysis (CGSEA) identified multiple candidate OA-associated genes and chemical compounds, such as Corticotropin Releasing Hormone Receptor 1 (*CRHR1*), Latent Transforming Growth Factor Beta Binding Protein 1 (*LTBP1*), WW Domain Containing E3 Ubiquitin Protein Ligase 2 (*WWP2*), LIM Homeobox Transcription Factor 1 Beta (*LMX1B*), and Parathyroid Hormone Like Hormone (*PTHLH*) [29]. Analyses of human cartilage tissues revealed that OA severity is significantly associated with reduced expression levels of Transforming Growth Factor Beta Receptor 2 (*TGFBR2*) and Interleukin 36 Receptor Antagonist (*IL-36RN*), while IL-36α expression exhibits an increasing trend [30].

Although accumulating evidence suggests that DNA methylation plays an important role in the development and progression of OA, systematically identifying key methylation biomarkers and their potential regulatory genes remains challenging. In this study, we aimed to develop a multidimensional computational framework integrating statistical analysis, machine learning, deep learning, and functional genomics based on DNA methylation data. Within this framework, a deep learning-based fusion model was developed to effectively screen for OA-associated DNA methylation biomarkers. Based on these biomarkers, several previously reported effector genes closely related to OA pathogenesis were identified, while additional novel potential candidate genes were further uncovered.

## 2. Results

### 2.1. Preliminary CpG Site Screening Based on Dual Strategies

**Figure 1:**
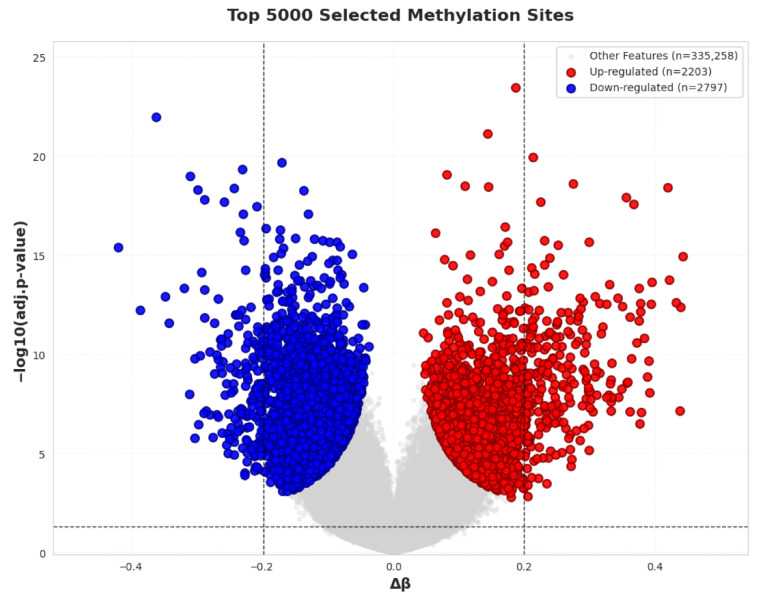
Volcano plot of CpG sites selected based on differential methylation analysis. This figure illustrates the differential DNA methylation patterns of 5000 CpG sites preliminarily screened using a combination of *t*-test and mean difference in methylation levels (Δβ). Red dots represent hypermethylated CpG sites, blue dots indicate hypomethylated CpG sites, and gray dots denote CpG sites that did not meet the selection criteria. Among the selected sites, 2203 CpG sites exhibited increased methylation levels, while 2797 CpG sites showed decreased methylation levels.

**Figure 2:**
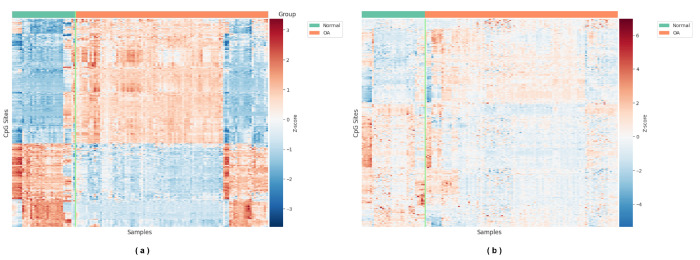
Heatmap visualization of differentially methylated CpG sites in T-pools and G-pools. Normal represents healthy control samples, and OA represents osteoarthritis samples. Red indicates higher standardized methylation levels, while blue indicates lower standardized methylation levels. (**a**) Heatmap of 412 CpG sites from T-pools that met both statistical and biological significance criteria (adj.*p*-value <0.05 and |Δβ|>0.2). (**b**) Heatmap of 412 representative CpG sites selected from G-pools.

The volcano plot of the feature subset T-pools, obtained through differential methylation analysis, is shown in Figure 1. Among the selected CpG sites, 2203 sites exhibited increased methylation levels, while 2797 sites showed decreased methylation levels. Within T-pools, a total of 412 CpG sites met both statistical and biological significance criteria (adj.*p*-value <0.05 and |Δβ|>0.2), and their methylation patterns are visualized in the heatmap shown in Figure 2a. For the feature subset G-pools, which was derived by selecting CpG sites located within ±2000 bp of the transcription start sites (TSSs) of OA effector genes, representative CpG site methylation patterns are displayed in the heatmap shown in Figure 2b.

### 2.2. Feature Selection Based on Machine Learning

**Figure 3:**
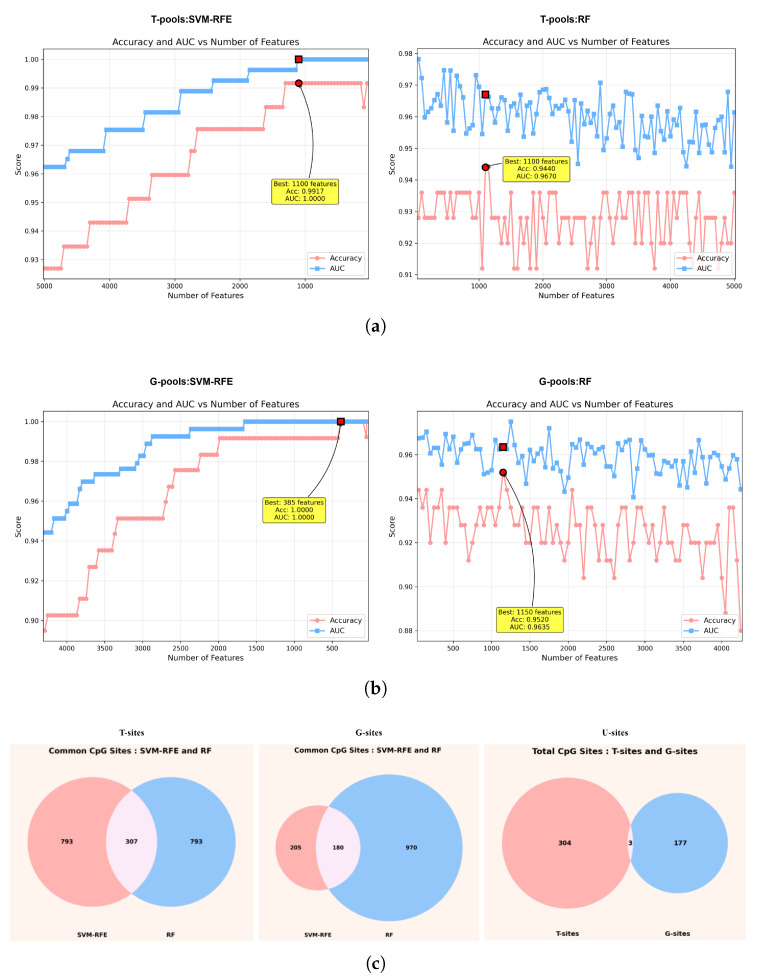
Feature selection using machine learning models. (**a**) Identification of CpG sites from T-pools using SVM-RFE and RF. (**b**) Identification of CpG sites from G-pools using SVM-RFE and RF. (**c**) Integration of T-sites and G-sites to generate the unified CpG site set (U-sites).

The results of feature selection for T-pools and G-pools using two machine learning models are shown in Figure 3. For T-pools, both the Support Vector Machine–Recursive Feature Elimination (SVM-RFE) and RF models each selected an optimal subset of 1100 CpG sites (Figure 3a). The intersection of these two subsets contained 307 CpG sites, which were defined as the dataset T-sites (Figure 3c). For G-pools, the SVM-RFE and RF models selected optimal feature subsets of 385 and 1150 CpG sites, respectively (Figure 3b). Their intersection consisted of 180 CpG sites, defined as G-sites (Figure 3c). Further analysis revealed that only three CpG sites overlapped between the T-sites and G-sites, indicating that the statistically driven features derived from differential methylation analysis and the biologically driven features based on prior knowledge of effector genes are highly complementary. These two feature sets capture OA-related methylation signals from distinct perspectives. To fully exploit this complementary information, we took the union of the two sets, resulting in an integrated feature set comprising 484 CpG sites, which was designated as U-sites (Figure 3c).

### 2.3. Diagnostic Performance Comparison of Different Feature Sets

**Figure 4:**
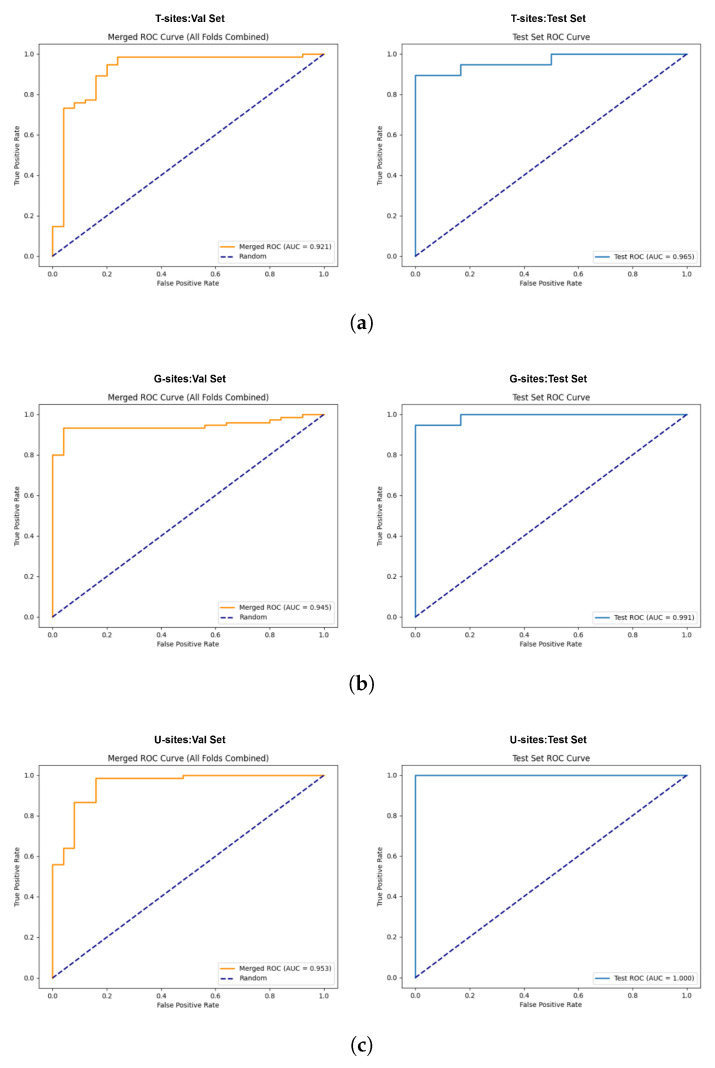
Diagnostic performance of the Trans-CNN model. (**a**) ROC curve of the diagnostic model constructed based on T-sites. (**b**) ROC curve of the diagnostic model constructed based on G-sites. (**c**) ROC curve of the diagnostic model constructed based on U-sites.

A comparison of the diagnostic performance metrics of the three feature sets using the Trans-CNN model is presented in Table 1. On the validation set, the model trained on U-sites achieved the best diagnostic performance, with an Accuracy (ACC) of 0.9400, an F1-score (F1) of 0.9613, and an Area Under the Receiver Operating Characteristic Curve (AUC) of 0.9530, outperforming the models trained on T-sites (ACC = 0.9100, AUC = 0.9210) and G-sites (ACC = 0.9300, AUC = 0.9450). On the independent test set, the U-sites-based model also demonstrated superior performance, achieving consistently strong results across all evaluation metrics, with ACC, F1-score, and AUC all reaching 1.0000. In comparison, the test-set ACC of the models trained on T-sites and G-sites were 0.9200 and 0.9600, respectively. The Receiver Operating Characteristic (ROC) curve curves of the three datasets on both the validation and test sets are presented in Figure 4.

These results indicate that the U-sites dataset, constructed by integrating statistically driven features (T-sites) and biologically informed features (G-sites), is able to capture OA-related methylation variation more comprehensively, thereby substantially improving the diagnostic performance of the model. Consequently, these findings provide strong evidence for the complementarity between the feature sets derived from the two pathways. Meanwhile, the U-sites dataset was selected as the candidate dataset for screening OA biomarkers in this study.

### 2.4. Biomarker

**Figure 5:**
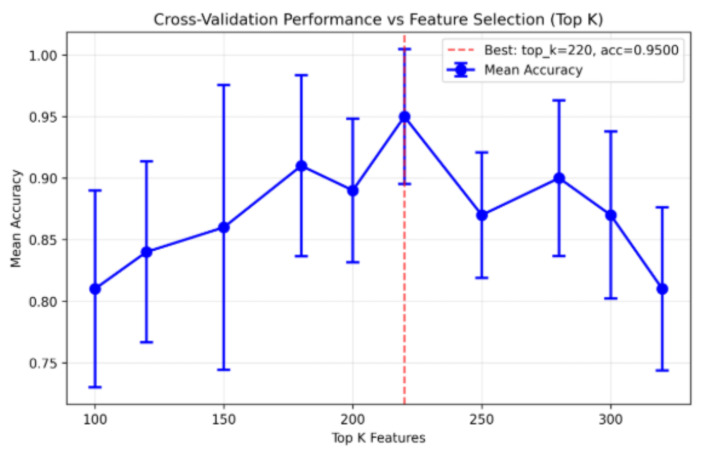
Comparison of Model Performance Across Different U-sites Feature Sets. The model achieved optimal performance when trained using the top 220 ranked features.

As shown in Figure 5, model performance exhibited a trend of initially increasing and then gradually declining with the increase in the number of features. When the top 220 ranked CpG sites were used, the validation ACC reached its maximum value of 0.9500, surpassing the performance obtained using the full U-sites dataset (484 CpG sites). Based on this optimization result, these 220 CpG sites were selected as the candidate biomarker set and designated as U-220.

**Figure 6:**
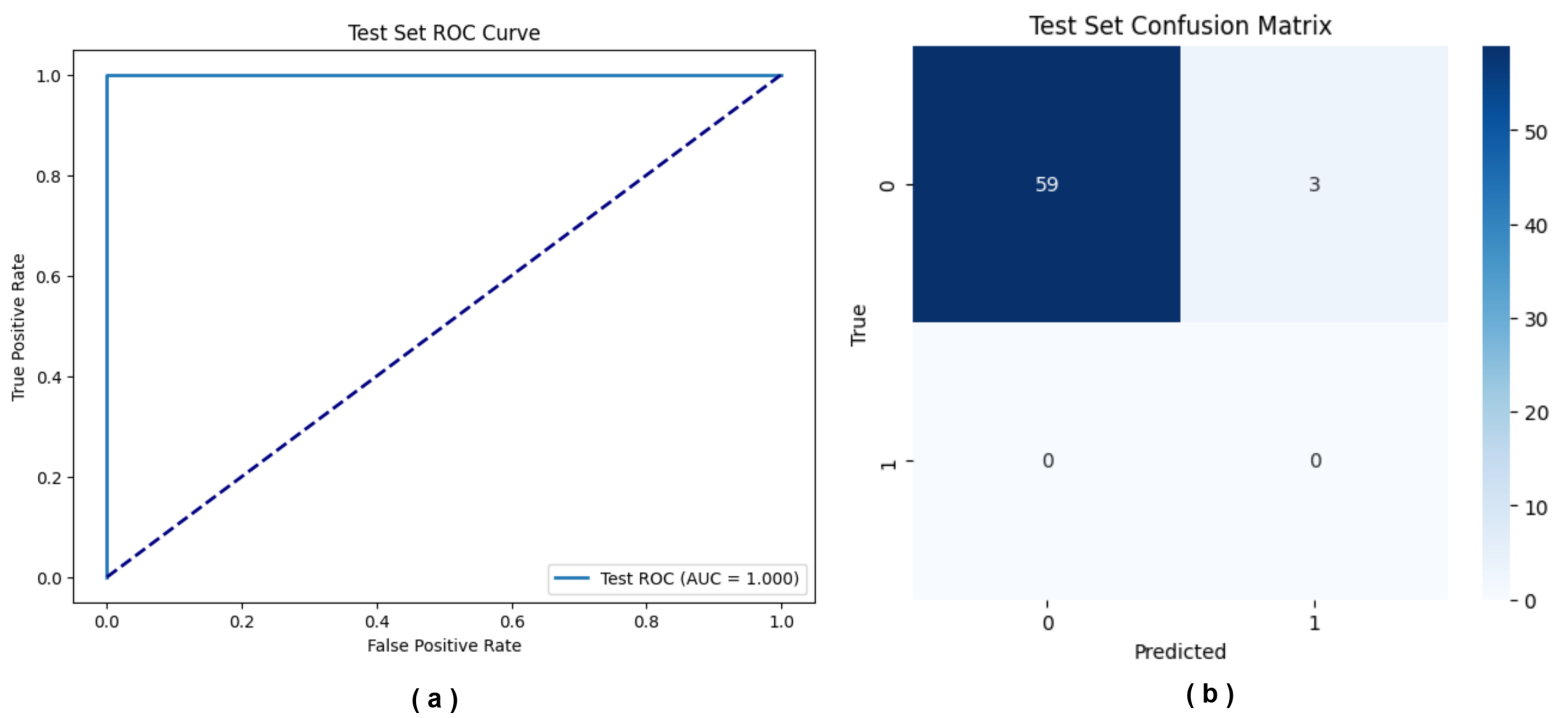
Diagnostic Performance of U-220. (**a**) ROC curve on the internal test set. (**b**) Confusion matrix of classification results on the independent external test set GSE63106.

On the internal test set, the model trained on the U-220 feature set achieved excellent classification performance, with ACC, F1-score, and AUC all reaching 1.00 (Table 1). The corresponding ROC curve is shown in Figure 6, with an AUC of 1.00, indicating that the model can perfectly distinguish between osteoarthritis patients and healthy controls.

To further evaluate the model’s generalization performance, external validation was conducted on the GSE63106 dataset, which includes 62 osteoarthritis samples without healthy controls. Although this dataset does not allow assessment of false positive predictions in healthy individuals or specificity-related metrics, it reflects the model’s ability to identify disease samples. The results show that the model achieved an ACC of 0.9516 on GSE63106, with the confusion matrix presented in Figure 6. Among the 62 osteoarthritis samples, 59 were correctly identified, with only 3 misclassified cases. These findings provide supportive evidence for the robustness of the U-220 feature set combined with the Trans-CNN model in capturing disease-associated patterns. In summary, the U-220 feature set was ultimately selected as a panel of DNA methylation biomarkers for osteoarthritis in this study.

### 2.5. Composition and Model Contribution of the U-220 Biomarker Panel

**Figure 7:**
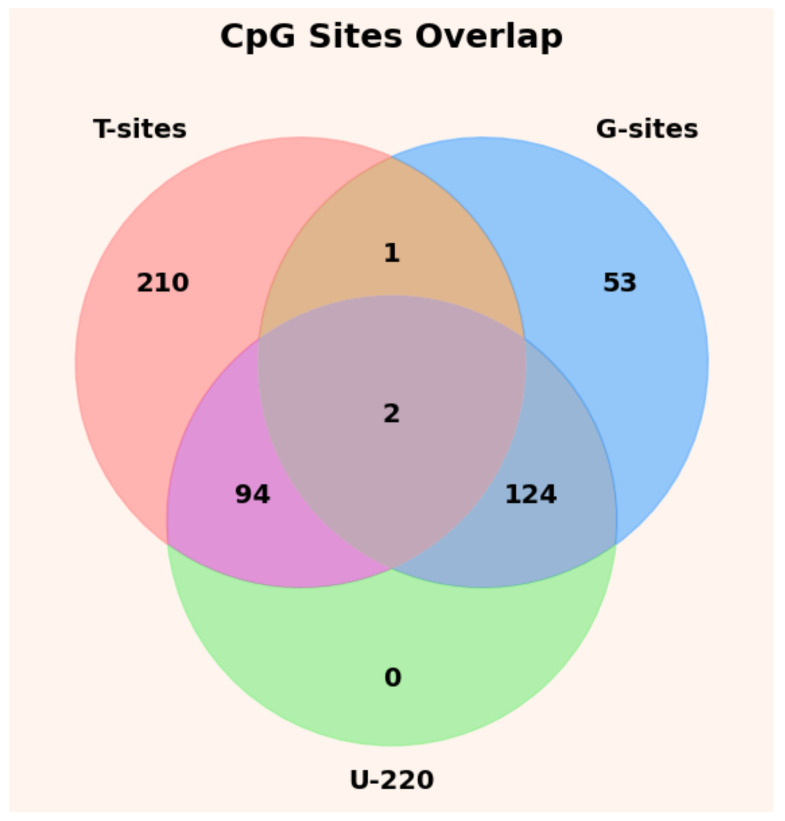
Analysis of the Origins of U-220 Sites. Two CpG sites were shared between T-sites and G-sites; in this study, these overlapping sites were classified as originating from G-sites.

As shown in Figure 7, the U-220 CpG site set is primarily derived from two distinct feature selection strategies: 94 sites originate from T-sites (denoted as T-94), while the remaining 126 sites are derived from G-sites (denoted as G-126). In terms of overall composition, T-94 and G-126 account for 42.7% and 57.3% of the U-220 set, respectively, indicating that biologically informed prior knowledge plays a relatively dominant role in the construction of the candidate biomarker panel. These findings underscore the importance of incorporating prior biological knowledge into the feature selection process.

**Figure 8:**
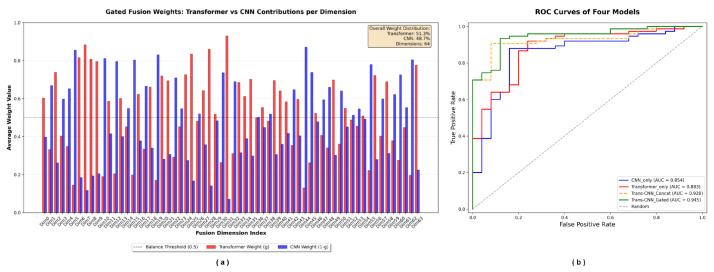
Importance Analysis of the Diagnostic Model. (**a**) Contribution analysis of features extracted by the two modules, with the average contribution weights of Transformer- and CNN-extracted features being 51.3% and 48.7%, respectively. (**b**) Comparative experiments among single-branch Transformer, single-branch CNN, dual-branch simple feature concatenation, and the diagnostic model proposed in this study. The results demonstrate that the diagnostic model developed herein achieves the best performance.

To further assess the relative importance of the two deep learning modules in model decision-making, we performed a statistical analysis of the distribution of the gating vector *g* obtained from the model trained on the U-220 dataset. As shown in Figure 8a, during the feature fusion stage, the average contribution weights of features extracted by the Transformer and CNN modules were 51.3% and 48.7%, respectively, demonstrating an approximately balanced distribution. These results validate the substantial complementarity between the two feature extraction mechanisms, with both contributing nearly equally to model decisions, indicating that the multi-branch architecture effectively captures feature patterns at different scales.

From a model design perspective, the dynamic gating mechanism successfully achieves balanced utilization of the two types of features. To further verify the superior performance of our constructed model, comparative experiments were conducted among a single-branch Transformer, a single-branch CNN, a dual-branch model with simple concatenation, and our proposed diagnostic model, as illustrated in Figure 8b. The results indicate that our diagnostic model adaptively modulates feature contributions at the dimensional level through learned gating weights, thereby maximizing the synergistic advantage of the heterogeneous network architecture and enhancing classification and diagnostic performance.

In summary, the feature datasets obtained through two distinct selection strategies were integrated and optimized using a diagnostic model capable of effectively capturing multi-scale biological information. The resulting U-220 biomarker panel exhibits both methodological complementarity and high reliability.

### 2.6. Enrichment Analysis

#### 2.6.1. Gene Mapping of U-220 CpG Sites

The CpG site combination U-220 was mapped to 180 genes based on Illumina Infinium HumanMethylation450 BeadChip (450K) annotations. Among these genes, 91 have been previously validated as OA effector genes in the study titled “Translational genomics of osteoarthritis in 1,962,069 individuals”. Of the remaining 89 genes, 41 have been reported as effector genes related to osteoarthritis in the Multi-omics Molecular Biomarkers and Database of Osteoarthritis (OAOB) or in recent related studies. The remaining 48 genes were considered potential genes related to osteoarthritis that require further validation.

#### 2.6.2. GO Enrichment Analysis

**Figure 9:**
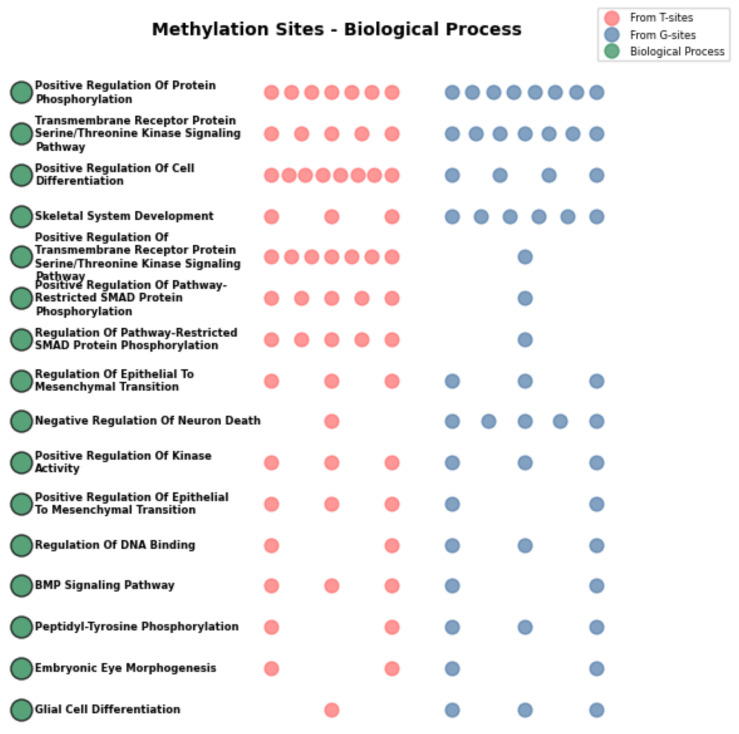
GO enrichment analysis of U-220 CpG site-mapped genes. The figure displays the enriched biological processes and the respective counts of CpG sites originating from distinct sources.

Gene Ontology (GO) enrichment analysis of the 180 genes revealed that CpG sites derived from T-94 and G-126 jointly enriched 16 biological processes, as shown in Figure 9. Among these, several biological processes—including the Bone Morphogenetic Protein (BMP) signaling pathway, positive regulation of transmembrane receptor protein serine/threonine kinase signaling, regulation of pathway-restricted SMAD protein phosphorylation, skeletal system development, and regulation of Epithelial to Mesenchymal Transition (EMT)—have been closely associated with the pathogenesis of OA [31,32,33,34,35]. As illustrated in the figure, genes originating from T-sites were primarily involved in biological processes such as positive regulation of protein phosphorylation, positive regulation of cell differentiation, and positive regulation of transmembrane receptor protein serine/threonine kinase signaling. Collectively, these results indicate that U-220 not only possesses diagnostic value from a statistical perspective but also exhibits a clear basis in biological function.

#### 2.6.3. WikiPathways Enrichment Analysis

**Figure 10:**
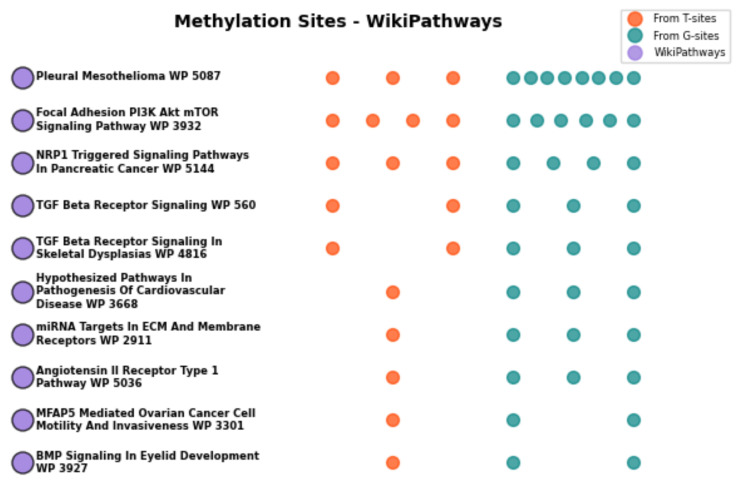
WikiPathways enrichment analysis of U-220 CpG site-mapped genes. The figure displays the enriched pathways and the respective counts of CpG sites originating from distinct sources.

To further elucidate the functional implications of the 180 genes, WikiPathways enrichment analysis was conducted, with the results illustrated in Figure 10. The enriched pathways were primarily implicated in the Transforming Growth Factor-β (TGF-β) /BMP signaling axis, microRNA (miRNA) targets, extracellular matrix organization, and phosphatidylinositol 3-kinase (PI3K)–protein kinase B (Akt)-mediated mechanotransduction [36,37,38,39]. Collectively, these pathways are recognized as critical regulators of cartilage homeostasis, chondrocyte differentiation, and osteophyte formation, thereby underscoring the biological significance of the identified DNA methylation biomarkers in the pathogenesis of OA.

#### 2.6.4. Circular Visualization of Enrichment Analysis

**Figure 11:**
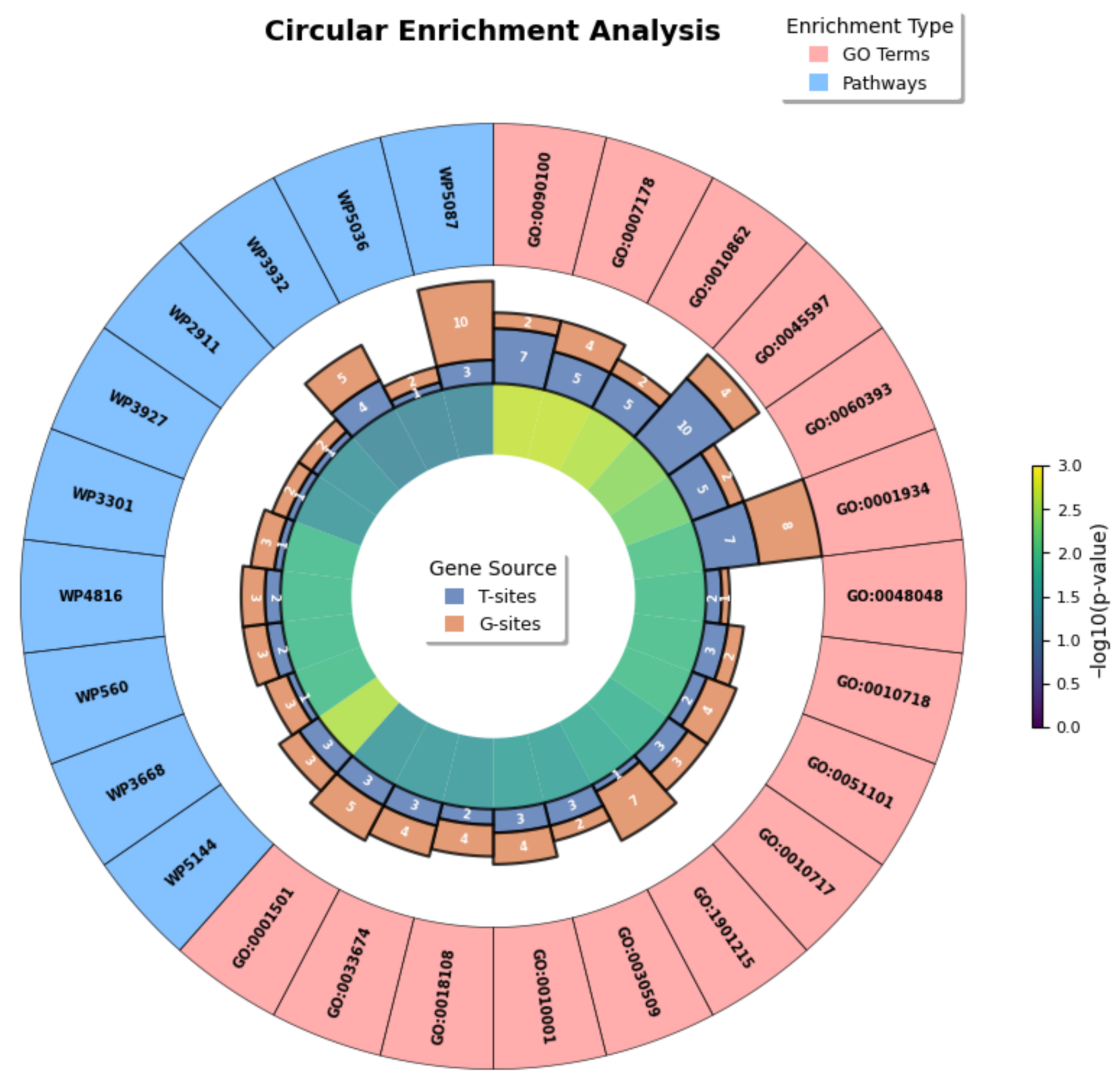
Circular visualization of dual enrichment analysis.

To comprehensively characterize the functional relevance of the biomarker set (U-220), a circular visualization was constructed to present the enrichment results (Figure 11). The outermost ring represents the identifiers of enriched GO biological processes and WikiPathways, while the middle ring shows the number of genes contributing to each term, categorized according to their origins within the U-220 dataset (T-sites and G-sites). The innermost ring reflects the enrichment significance, expressed as $-\log_{10}$(*p*-value).

As illustrated in the figure, the enriched terms are predominantly associated with signal transduction, extracellular matrix organization, and cell differentiation, all of which are well-recognized as key mechanisms underlying the pathogenesis of OA. Notably, in the majority of OA-related biological processes and pathways, genes derived from T-sites constitute a substantial proportion of the contributing genes. This observation suggests that T-site–associated genes may play an important role in driving these functional alterations. Therefore, these findings not only validate the biological functionality of U-220 as a biomarker but also provide a reference direction for the subsequent screening of potential key genes associated with OA.

### 2.7. PPI Network Analysis and Key Gene Identification

**Figure 12:**
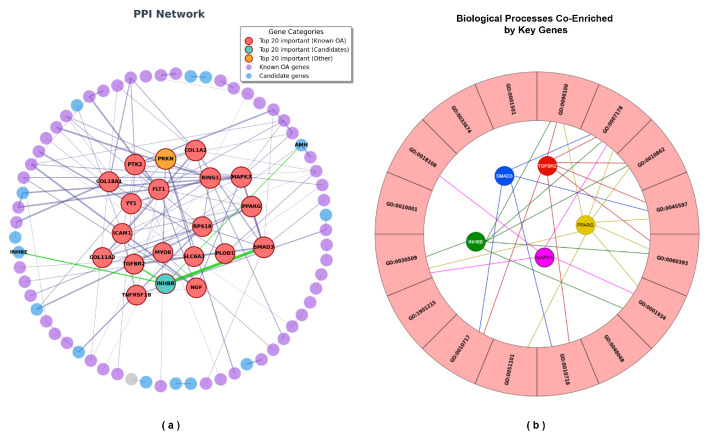
PPI analysis of proteins encoded by U-220 mapped genes and visualization of biological processes involving five key genes in U-220 enrichment. (**a**) PPI network of 180 genes mapped from the U-220 CpG site set. In the outer layer, purple nodes represent previously validated OA effector genes, blue nodes represent genes not yet validated, and gray nodes indicate filler nodes. The inner layer highlights the top 20 genes with the most significant interactions in the PPI network: red nodes indicate validated OA effector genes, green nodes denote candidate key genes pending validation, and yellow nodes represent filler nodes. Gray edges indicate PPI, with edge thickness reflecting the interaction significance; thicker edges correspond to more significant interactions. Green edges specifically highlight interactions involving the *INHBB* node. (**b**) Visualization of BP enriched by U-220 genes, highlighting the participation of five key genes. Red nodes represent *TGFBR2*, blue nodes represent *SMAD3*, yellow nodes represent *PPARG*, purple nodes represent *MAPK3*, and green nodes represent *INHBB*. The corresponding colored edges indicate the biological processes in which each key gene is involved.

Protein–protein interaction (PPI) network analysis was performed on the proteins encoded by the 180 genes mapped from U-220, identifying 182 proteins and 116 significant interaction edges. The PPI network is presented in Figure 12a, where the inner layer highlights the top 20 key proteins exhibiting the most significant interactions. Among these 20 genes, 18 have been previously validated as OA effector genes, one gene (*INHBB*) represents a potential OA key gene requiring further validation, and one node corresponds to an automatically supplemented interacting gene. Topological analysis of the PPI network revealed that *SMAD3*, *PPARG*, and *MAPK3* ranked as the top three in terms of composite scores, with strong interactions observed among them. Additionally, *TGFBR2* exhibited the most significant interaction with *SMAD3* among all nodes interacting with *SMAD3*. In OA research, *TGFBR2*, *PPARG*, *MAPK3*, and *SMAD3* have been well established as key regulators involved in the critical mechanisms of OA pathogenesis. PPI analysis demonstrated that *INHBB* directly interacts with *TGFBR2* and *SMAD3*, suggesting its involvement in core signaling networks associated with osteoarthritis.

Combined with the enrichment analysis of the 180 genes, five genes—*TGFBR2*, *PPARG*, *MAPK3*, *SMAD3*, and *INHBB*—were found to be co-enriched in the biological process of the Transmembrane Receptor Protein Serine/Threonine Kinase Signaling Pathway (GO:0007178), as illustrated in Figure 12b. This process involves the phosphorylation of specific serine or threonine residues, directly and cross-regulating downstream signaling cascades, including the TGF-β/BMP signaling, TGF-β/Smad pathway, Wnt/β-catenin signaling pathway, and NF-κB signaling pathway, all of which are closely associated with OA pathogenesis [40,41].

In conclusion, the PPI network and enrichment analyses indicate that *TGFBR2*, *PPARG*, *MAPK3*, and *SMAD3* play central regulatory roles in the molecular networks associated with osteoarthritis, while *INHBB* emerges as a novel potential key gene for OA with significant network connectivity. Therefore, *TGFBR2*, *PPARG*, *MAPK3*, *SMAD3*, and *INHBB* were identified as key genes involved in the regulatory mechanisms of OA, while *AMH* and *INHBE*, which exhibit significant interactions with *INHBB*, may also participate in these regulatory mechanisms and exert functional roles.

### 2.8. KEGG Pathway Mapping of Key Genes

**Figure 13:**
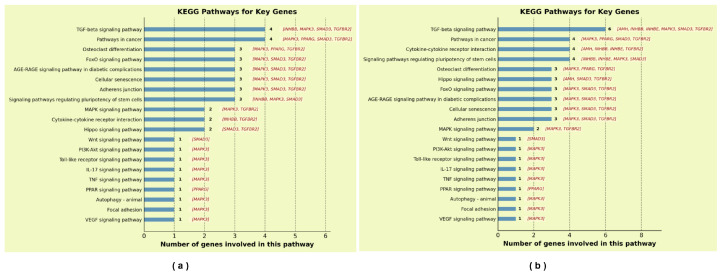
KEGG pathway mapping. (**a**) Pathway mapping of five key genes. (**b**) Pathway mapping of seven genes.

The mapping results of the genes in the KEGG pathways are presented in Figure 13. Kyoto Encyclopedia of Genes and Genomes (KEGG) pathway mapping of the five key genes revealed that both the TGF-β signaling pathway and Pathways in cancer contained four mapped genes. Specifically, the TGF-β signaling pathway included *TGFBR2*, *SMAD3*, *MAPK3*, and *INHBB*, while Pathways in cancer comprised *TGFBR2*, *SMAD3*, *MAPK3*, and *PPARG*. In addition, several pathways closely associated with OA pathogenesis—including osteoclast differentiation, FoxO signaling pathway, AGE–RAGE signaling pathway in diabetic complications, cellular senescence, adherens junction, and signaling pathways regulating pluripotency of stem cells—each contained three mapped genes. Notably, *INHBB* was involved in the signaling pathways regulating pluripotency of stem cells. *TGFBR2*, *SMAD3*, and *MAPK3* were frequently co-mapped across multiple OA-related pathways, consistent with their well-established central regulatory roles in OA. As a newly identified candidate gene in this study, *INHBB* was co-mapped with these three known OA effector genes in the TGF-β signaling pathway, suggesting its potential involvement in OA pathogenesis through interaction with the TGF-β/Smad signaling network.

To further investigate genes interacting with *INHBB*, *AMH* and *INHBE* were incorporated into the analysis, and KEGG pathway mapping was performed on the expanded gene set. The results showed that the number of genes mapped to the TGF-β signaling pathway increased from four to six, with *AMH* and *INHBE* newly included. Meanwhile, the cytokine–cytokine receptor interaction pathway and signaling pathways regulating pluripotency of stem cells each contained four mapped genes, all including *AMH*, *INHBB*, and *INHBE*. Notably, the inclusion of *AMH* and *INHBE* expanded the representation of genes within the TGF-β signaling pathway.As interacting partners of *INHBB*, *AMH* and *INHBE* were co-mapped with *INHBB* in both the TGF-β signaling pathway and cytokine–cytokine receptor interaction pathway, suggesting that they may cooperatively participate in OA pathogenesis through the TGF-β/Smad signaling network and inflammation-related pathways.

In summary, in addition to the well-established OA effector genes *TGFBR2*, *PPARG*, *MAPK3*, and *SMAD3*, *INHBB* was identified as a novel key gene associated with OA. Furthermore, *AMH* and *INHBE*, which exhibit strong network connectivity with *INHBB* and are co-mapped in key pathways, are proposed as potential OA-related genes warranting further functional validation.

## 3. Discussion

In this study, we developed a multidimensional computational framework integrating statistical analysis, machine learning, deep learning, and functional genomics to identify key genes involved in OA pathogenesis. Our analysis confirmed the central regulatory roles of known OA effector genes—*TGFBR2*, *SMAD3*, *PPARG*, and *MAPK3*—while identifying *INHBB* as a novel key gene with significant network connectivity. Furthermore, *AMH* and *INHBE* were proposed as potential OA-related genes that may function synergistically with *INHBB*. These findings provide new insights into the molecular mechanisms underlying OA and offer potential targets for further functional validation.

First, our integrative analysis supports the hypothesis that DNA methylation signatures can reflect biologically meaningful regulatory mechanisms underlying OA. The mapping of U-220 to 180 genes revealed that a substantial proportion (over 70%) have already been reported as OA effector genes, either in large-scale translational genomics studies or curated OA databases. This high overlap strongly validates the biological relevance of our feature selection strategy and supports the hypothesis that methylation-derived biomarkers are not merely predictive but mechanistically informative. Previous studies have shown that epigenetic alterations, particularly DNA methylation, play a critical role in modulating gene expression involved in cartilage degradation and inflammation in OA [11].

GO enrichment analysis further demonstrated that these genes are significantly involved in biological processes tightly linked to OA pathogenesis, particularly the BMP signaling pathway, positive regulation of transmembrane receptor serine/threonine kinase signaling, and SMAD protein phosphorylation [31,32,33]. These processes are central components of the TGF-β/BMP signaling axis, which is known to regulate chondrocyte differentiation, ECM homeostasis, and cartilage repair. Dysregulation of TGF-β signaling has been widely reported to contribute to cartilage degeneration and osteophyte formation in OA [32]. Moreover, the enrichment in EMT-related processes is consistent with emerging evidence that chondrocyte phenotypic shifts resemble EMT-like transitions during OA progression [35]. These findings collectively confirm that our identified CpG sites are functionally enriched in biologically meaningful pathways relevant to OA, thereby supporting our initial hypothesis.WikiPathways analysis extended these findings by highlighting key signaling axes, including TGF-β/BMP, PI3K-Akt, and miRNA-mediated regulation. The PI3K-Akt pathway has been shown to mediate mechanotransduction and regulate chondrocyte survival and metabolism under mechanical stress conditions [39]. Similarly, miRNAs are well-established post-transcriptional regulators in OA, modulating inflammation, apoptosis, and ECM degradation [37]. The convergence of these pathways suggests that the identified gene set participates in a coordinated regulatory network controlling cartilage homeostasis and OA progression.

PPI network analysis further identified *SMAD3*, *PPARG*, and *MAPK3* as central hub genes, with strong interaction connectivity. These genes are well-known regulators in OA: *SMAD3* is a key mediator of canonical TGF-β signaling and has been genetically associated with OA susceptibility [28]; *MAPK3* is involved in MAPK signaling, contributing to inflammatory responses and cartilage catabolism [28,42]; and *PPARG* plays an anti-inflammatory role and regulates lipid metabolism and chondrocyte differentiation [23,43]. Importantly, *TGFBR2*, a receptor upstream of SMAD signaling, showed strong interaction with *SMAD3*, reinforcing the central role of the TGF-β/Smad axis in OA [30]. Notably, *INHBB* emerged as a novel candidate gene with strong connectivity to *TGFBR2* and *SMAD3*. As a member of the TGF-β superfamily, *INHBB* encodes a subunit of activin proteins, which are known to regulate cell proliferation and differentiation [44]. While INHBB has not been previously implicated in OA, studies in other inflammatory and degenerative conditions have suggested its involvement in tissue remodeling and inflammatory responses [45]. The co-enrichment of *INHBB* with established OA genes in the TGF-β signaling pathway, along with its direct interactions in the PPI network, strongly suggests that it may contribute to OA pathogenesis through modulation of the TGF-β/Smad signaling cascade.

KEGG pathway mapping provided additional support for this conclusion. The frequent co-mapping of *TGFBR2*, *SMAD3*, and *MAPK3* across multiple OA-related pathways—such as osteoclast differentiation, FoxO signaling, and cellular senescence—highlights their pleiotropic regulatory roles in cartilage degeneration and aging-related processes [46,47,48]. Importantly, *INHBB* was co-mapped with these genes in the TGF-β signaling pathway, further supporting its integration into this core regulatory network. To further explore the functional network surrounding *INHBB*, we extended our analysis to include its interacting partners *AMH* and *INHBE*. *AMH* is best known for its role in sexual differentiation, but recent evidence suggests its involvement in inflammatory modulation and tissue homeostasis. *INHBE* encodes the inhibin beta E subunit, which, like *INHBB*, can form activin complexes and has been implicated in metabolic regulation and inflammatory responses [49,50]. Our KEGG pathway analysis revealed that the inclusion of *AMH* and *INHBE* expanded the representation of genes in the TGF-β/signaling pathway from four to six, and these three genes were co-enriched in the cytokine-cytokine receptor interaction pathway. These findings suggest that AMH and INHBE may act cooperatively with *INHBB* to modulate inflammatory and signaling processes relevant to OA.

Despite the successful identification of key genes in this study, several limitations should be acknowledged. First, the imbalance in the dataset limits the validation of the model’s diagnostic generalizability to some extent. Second, the findings rely on public databases and in silico predictions, lacking experimental validation. Third, the cross-sectional nature of the methylation data precludes causal inference regarding the role of these genes in OA progression.Future studies should focus on experimental validation of *INHBB* and its interacting partners (*AMH* and *INHBE*) in OA models, particularly investigating their roles in TGF-β//Smad signaling and cartilage homeostasis. Multi-omics integration, including transcriptomics and proteomics, could further elucidate the regulatory mechanisms linking DNA methylation to gene expression and protein activity.

In summary, our study demonstrates that the CpG biomarker set U-220 captures key molecular features of OA and identifies *INHBB* as a novel candidate gene potentially involved in the TGF-β/-centered regulatory network. These findings provide new insights into the epigenetic regulation of OA and offer a foundation for future mechanistic and translational research.

## 4. Materials and Methods

### 4.1. Data Sources and Preprocessing

In this study, DNA methylation datasets related to OA were retrieved from the Gene Expression Omnibus (GEO) database using “osteoarthritis” as the search keyword. Three datasets, GSE63695, GSE73626, and GSE162484, were selected as the training datasets, comprising 94 OA patient samples and 31 healthy control samples. Additionally, the GSE63106 dataset, which includes 62 OA samples, was employed as an independent external test dataset.All four datasets were generated using the 450K platform, which provides standardized labeling of patient and control samples. In the training datasets, 73 OA samples were derived from the knee joint and 21 OA samples from the hip joint. Among the healthy controls, 5 samples were obtained from the knee joint and 26 samples from the hip joint. In the independent external test dataset GSE63106, OA patient samples included 34 knee joint samples and 28 hip joint samples.As this study focused on overall differences between OA patients and healthy controls, OA samples were labeled as 1, whereas healthy control samples were labeled as 0. All datasets underwent a standardized preprocessing pipeline, including selection of CpG sites common to all datasets, removal of sites containing missing values, batch effect correction, standardization of numerical precision, and genomic ordering of CpG sites according to chromosomal positions.

### 4.2. Preliminary CpG Site Screening

After preprocessing, we obtained an original dataset comprising a total of 340,258 DNA methylation sites. Preliminary screening of CpG sites was performed through two independent approaches. The first approach involved differential methylation analysis, specifically *t*-test and analysis of the mean difference in methylation levels between the two groups. Here, the *p*-value from the *t*-test adjusted by the false discovery rate (FDR) is denoted as adj. *p*-value, and the mean difference in methylation levels between the two groups is denoted as Δβ. CpG sites were ranked based on statistical significance (adj. *p*-value) and biological significance (Δβ), and the top 5000 sites were selected as a feature subset, referred to as T-pools. In the second approach, CpG sites located within ±2000 bp of TSSs of known OA effector genes were selected. A total of 700 OA-related effector genes reported in the study “Translational genomics of osteoarthritis in 1,962,069 individuals” were used as reference genes. Based on this criterion, 4291 CpG sites were identified in the second approach and defined as another feature subset, referred to as G-pools.

### 4.3. Feature Selection Based on Machine Learning

The feature subsets from the T-pool and G-pool were independently input into the SVM-RFE and RF models. In both models, the response variable was defined as the disease status of each sample (OA vs. Normal), serving as the class label for supervised learning. For each model, five-fold cross-validation was performed, and the mean ACC and AUC were recorded. Within both the SVM-RFE and RF frameworks, the selection of CpG site combinations was primarily based on ACC; when ACC values were identical, AUC was used as a secondary selection criterion. CpG sites commonly selected by both SVM-RFE and RF were retained as robust features for downstream diagnostic modeling. Specifically, CpG sites jointly identified from the T-pool by the two models were defined as T-sites, whereas those jointly identified from the G-pool were defined as G-sites. Furthermore, the union of T-sites and G-sites was designated as U-sites, representing an integrated CpG feature set for subsequent model construction.

### 4.4. Construction of the Diagnostic Model

**Figure 14:**
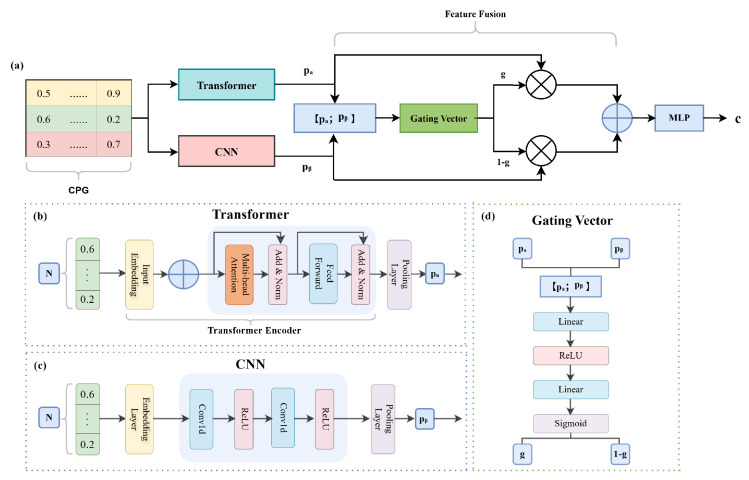
Schematic overview of the Trans-CNN diagnostic model. (**a**) Overall architecture of the proposed Trans-CNN framework for DNA methylation-based OA classification. (**b**) Transformer module for modeling global dependencies among CpG sites. (**c**) CNN module for extracting local methylation patterns. (**d**) Gating mechanism for generating an adaptive gating vector for feature modulation.

The DNA methylation data were first mapped via an embedding layer and then processed in parallel by a Transformer encoder and a CNN model. Following global average pooling, the extracted features were fed into a dynamic weight-gated module for feature fusion at the same dimensionality, and subsequently into a classifier for final prediction. The overall architecture of the Trans-CNN diagnostic model is illustrated in Figure 14. The dynamic weight-gated feature fusion procedure was implemented as follows:

The Transformer encoder outputs a feature vector of shape (B,d_model), while the CNN outputs a feature vector of shape (B,cnn_out). Each feature vector was projected through a linear layer to obtain:

$$p_{a}\in R^{\left(B,\text{fusion\_dim}\right)},\;p_{\beta }\in R^{\left(B,\text{fusion\_dim}\right)}.$$

where *B* denotes the batch size, and d_model, cnn_out, and fusion_dim are the feature dimensions, all set to 64.

The projected features were concatenated and input into a neural network with two fully connected layers to generate a gating vector *g*, with elements ranging from 0 to 1. Each element of *g* represents the contribution weight of the features obtained from the Transformer, while 1−g corresponds to the contribution weight of the features obtained from the CNN. The formulation is as follows:

(1) $$\begin{array}{cc} Z & =[p_{a};p_{\beta }] \end{array}$$

(2) $$\begin{array}{cc} g & =\sigma (W_{2}\cdot \mathrm{ReLU}\left(W_{1}\cdot Z+b_{1}\right)+b_{2}) \end{array}$$

where $W_{1}$ and $W_{2}$ are learnable weight matrices, $b_{1}$ and $b_{2}$ are bias vectors, ReLU denotes the rectified linear unit activation function, σ denotes the sigmoid function, and $g\in R^{\left(B,\text{fusion\_dim}\right)}$.

Feature fusion was performed based on the gating vector:

(3) $$\mathrm{fused}=g⊙p_{a}+\left(1-g\right)⊙p_{\beta }$$

where ⊙ denotes element-wise multiplication. This mechanism enables adaptive weighting of Transformer and CNN features across different feature dimensions.

### 4.5. Diagnostic Performance Comparison of Different Feature Sets

To evaluate the impact of different feature sets on the diagnostic performance of the model, three datasets—T-sites (307 CpG sites), G-sites (180 CpG sites), and U-sites (484 CpG sites)—were used as inputs for training and evaluating the Trans-CNN diagnostic model. The classification performance was assessed using three metrics: ACC, F1-score, and AUC. These metrics are widely used to evaluate model performance in binary disease classification tasks [51]. The best-performing dataset was subsequently selected as the candidate dataset for biomarker identification.

### 4.6. Biomarker Selection

To further reduce feature dimensionality, decrease model complexity, and identify the most representative DNA methylation biomarkers while maintaining diagnostic performance, the 484 CpG sites in the U-sites dataset were ranked based on mean gradient importance. Feature subsets were then sequentially constructed by selecting CpG sites at regular intervals from the top-ranked positions, ranging from 100 to 320 (i.e., 100, 120, 150, 180, 200, 220, 250, 280, 300, and 320 features). For each subset, the Trans-CNN model was trained and the average classification ACC was recorded. By comparing the average ACC across different feature subsets, the subset achieving the highest ACC was selected as the candidate biomarker set. Finally, the diagnostic performance of the selected biomarkers was validated by comparing their performance metrics with those obtained using the full U-sites dataset.

### 4.7. Composition and Model Contribution Analysis of the Biomarker Panel

To systematically elucidate the compositional characteristics and potential biological background of the U-220 candidate biomarker set, we performed a source tracing and composition analysis of the 220 selected CpG sites. One of the core innovations of the Trans-CNN model lies in the introduction of a dynamic weight gating mechanism, which enables the adaptive integration of global dependency features extracted by the Transformer branch and local pattern features captured by the CNN branch. To further evaluate the relative importance of these two model components in feature selection and decision-making, we conducted a statistical analysis of the distribution of the gating vector *g* obtained from the model trained on the U-220 dataset.

### 4.8. Enrichment Analysis

To systematically elucidate the biological functions and underlying molecular regulatory networks of the U-220 biomarker panel, we performed GO enrichment analysis and WikiPathway enrichment analysis on the pgenes corresponding to the 220 CpG sites comprising this panel. In this study, multiple testing correction was performed using the Benjamini-Hochberg FDR procedure. Statistical significance was assessed based on FDR-adjusted *p*-values to rigorously control the false discovery rate.

### 4.9. PPI Network Analysis and Key Gene Identification

To investigate the PPI relationships among the proteins encoded by the 220 mapped genes, PPI analysis was performed on the 180 corresponding proteins using the STRING database. A minimum interaction confidence score threshold of 500 was applied to ensure the identification of interactions with high reliability. To evaluate the functional importance of each node within the network, two topological metrics—degree centrality and betweenness centrality—were integrated to rank the nodes. Degree centrality reflects the number of direct connections of a node, measuring its centrality within the local network, while betweenness centrality quantifies the frequency with which a node acts as a bridge along the shortest paths, indicating its role as a hub in information flow or signal transduction [52]. Following standardization, the two metrics were weighted and summed to obtain a comprehensive importance score for each node, and the top 20 critical nodes were selected based on this score.

### 4.10. KEGG Pathway Mapping of Key Genes

To evaluate the potential key role of INHBB in the pathogenesis of OA, KEGG pathway mapping was performed on the five key genes, and the results were visualized. Furthermore, to investigate the potential critical involvement of AMH and INHBE, KEGG pathway mapping was conducted on the seven genes, with the results visualized accordingly.

## 5. Conclusions

Based on DNA methylation data, this study successfully confirmed the central regulatory roles of known OA effector genes, including *TGFBR2*, *SMAD3*, *PPARG*, and *MAPK3*, within the molecular network. Additionally, the study identified *INHBB* as a novel potential key gene associated with OA. The proposed multidimensional computational framework provides an effective tool for mining OA effector genes from DNA methylation signals and offers new insights for investigating OA molecular mechanisms and facilitating clinical translation. In future studies, we will employ integrative multi-omics approaches to further conduct joint analyses of DNA methylation and gene expression, with the aim of systematically elucidating the molecular regulatory mechanisms underlying OA and enabling the precise identification of potential effector genes.

## Figures and Tables

**Table 1:** Performance comparison of the Trans-CNN model using T-sites, G-sites, U-sites, and U-220.

Dataset	Group	ACC	F1	AUC
T-sites	Val Set	0.9100 ± 0.0860	0.9387 ± 0.0554	0.9210
	Test Set	0.9200	0.9500	0.9650
G-sites	Val Set	0.9300 ± 0.0510	0.9548 ± 0.0353	0.9450
	Test Set	0.9600	0.9744	0.9910
U-sites	Val Set	0.9400 ± 0.0374	0.9613 ± 0.0270	0.9530
	Test Set	1.0000	1.0000	1.0000
U-220	Val Set	0.9500 ± 0.0316	0.9630 ± 0.0211	0.9590
	Test Set	1.0000	1.0000	1.0000

## Data Availability

All DNA methylation datasets used in this paper are from the Gene Expression Omnibus (GEO) database, which is a public comprehensive repository of gene expression data. All datasets are available at the following website: https://www.ncbi.nlm.nih.gov/geo/ (accessed on 1 September 2025).

## Abbreviations

The following abbreviations are used in this manuscript:

OA	Osteoarthritis
SMOADs	Symptom-modifying osteoarthritis drugs
DMOADs	Disease-modifying osteoarthritis drugs
SVM	Support Vector Machines
RF	Random Forest
CNN	Convolutional Neural Network
*ATOH8*	Atonal BHLH Transcription Factor 8
*MAFF*	MAF BZIP Transcription Factor F
*NCOR2*	Nuclear Receptor Corepressor 2
*TBX4*	T-Box Transcription Factor 4
*ZBTB16*	Zinc Finger And BTB Domain Containing 16
*ZHX2*	Zinc Fingers And Homeoboxes 2
DNMTs	DNA Methyltransferases
*ADAMTS-5*	A Disintegrin And Metalloproteinase With Thrombospondin Motifs 5
C/EBPα	CCAAT/Enhancer-Binding Protein Alpha
NF-κB	Nuclear Factor Kappa-B
*PPARG*	Peroxisome Proliferator Activated Receptor Gamma
GWAS	Genome-Wide Association Study
eQTL	Expression Quantitative Trait Loci
Hi-C	High-throughput Chromosome Conformation Capture
TWAS	Transcriptome-Wide Association Studies
FUSION	Functional summary-based imputation
*GCAT*	Glycine C-Acetyltransferase
*MAPK3*	Mitogen-Activated Protein Kinase 3
*MST1R*	Macrophage Stimulating 1 Receptor
*PFKM*	Phosphofructokinase, Muscle
*RAD9A*	RAD9 Checkpoint Clamp Component A
*SMAD3*	SMAD Family Member 3
*USP8*	Ubiquitin Specific Peptidase 8
CGSEA	Chemical Gene Set Enrichment Analysis
*CRHR1*	Corticotropin Releasing Hormone Receptor 1
*LTBP1*	Latent Transforming Growth Factor Beta Binding Protein 1
*WWP2*	WW Domain Containing E3 Ubiquitin Protein Ligase 2
*LMX1B*	LIM Homeobox Transcription Factor 1 Beta
*PTHLH*	Parathyroid Hormone Like Hormone
*TGFBR2*	Transforming Growth Factor Beta Receptor 2
*IL-36RN*	Interleukin 36 Receptor Antagonist
TSSs	Transcription Start Sites
SVM-RFE	Support Vector Machine–Recursive Feature Elimination
ACC	Accuracy
F1	F1-score
AUC	Area Under the receiver operating characteristic Curve
ROC	Receiver Operating Characteristic
GO	Gene Ontology
BMP	Bone Morphogenetic Protein
EMT	Epithelial to Mesenchymal Transition
TGF-β	Transforming Growth Factor-β
miRNA	microRNA
PI3K	phosphatidylinositol 3-kinase
PPI	Protein–Protein Interaction
KEGG	Kyoto Encyclopedia of Genes and Genomes
GEO	Gene Expression Omnibus
FDR	False Discovery Rate

## References

[1] Ferrari A.J., Santomauro D.F., Aali A., Abate Y.H., Abbafati C., Abbastabar H., Bell M.L. (2024). Global incidence, prevalence, years lived with disability (YLDs), disability-adjusted life-years (DALYs), and healthy life expectancy (HALE) for 371 diseases and injuries in 204 countries and territories and 811 subnational locations, 1990–2021: A systematic analysis for the Global Burden of Disease Study 2021. Lancet.

[2] Steinmetz J.D., Culbreth G.T., Haile L.M., Rafferty Q., Lo J., Fukutaki K.G., Singh S. (2023). Global, regional, and national burden of osteoarthritis, 1990–2020 and projections to 2050: A systematic analysis for the Global Burden of Disease Study 2021. Lancet Rheumatol..

[3] Zhang Y., Han Y., Sun Y., Hao L., Gao Y., Ye J., Wang H., Zhang T., Liu Y., Yang Y. (2026). Osteoarthritis: Molecular pathogenesis and potential therapeutic options. Sig. Transduct. Target. Ther..

[4] Chen D. (2022). Osteoarthritis: A complicated joint disease requiring extensive studies with multiple approaches. J. Orthop. Translat..

[5] Roemer F.W., Demehri S., Omoumi P., Link T.M., Kijowski R., Saarakkala S., Guermazi A. (2020). State of the art: Imaging of osteoarthritis—Revisited 2020. Radiology.

[6] Li J., Wang Y., Chen D., Liu-Bryan R. (2022). Oral administration of berberine limits post-traumatic osteoarthritis development and associated pain via AMP-activated protein kinase (AMPK) in mice. Osteoarthr. Cart..

[7] Brandt M.D., Malone J.B., Kean T.J. (2025). Advances and Challenges in the Pursuit of Disease-Modifying Osteoarthritis Drugs: A Review of 2010–2024 Clinical Trials. Biomedicines.

[8] Portela A., Esteller M. (2010). Epigenetic modifications and human disease. Nat. Biotechnol..

[9] Zhao L.-Y., Song J.H., Liu Y.B., Song C.-X., Yi C.Q. (2020). Mapping the epigenetic modifications of DNA and RNA. Protein Cell.

[10] Greenberg M.V.C., Bourc’his D. (2019). The diverse roles of DNA methylation in mammalian development and disease. Nat. Rev. Mol. Cell Biol..

[11] Nau T., Cutts S., Naidoo N. (2025). DNA methylation and its influence on the pathogenesis of osteoarthritis: A systematic literature review. EFORT Open Rev..

[12] Asif S., Wenhui Y., ur-Rehman S., ul-Ain Q., Amjad K., Yueyang Y., Awais M. (2025). Advancements and prospects of machine learning in medical diagnostics: Unveiling the future of diagnostic precision. Arch. Comput. Methods Eng..

[13] Lim A.J., Tyniana C.T., Lim L.J., Tan J.W.L., Koh E.T., Chong S.S., Lee C.G. (2023). Robust SNP-based prediction of rheumatoid arthritis through machine-learning-optimized polygenic risk score. J. Transl. Med..

[14] Lampe L., Niehaus S., Huppertz H.J., Merola A., Reinelt J., Mueller K., Schroeter M.L. (2022). Comparative analysis of machine learning algorithms for multi-syndrome classification of neurodegenerative syndromes. Alzheimer’s Res. Ther..

[15] Wu Z., Shou L., Wang J., Huang T., Xu X. (2020). The Methylation Pattern for Knee and Hip Osteoarthritis. Front. Cell Dev. Biol..

[16] AbdulAzeem Y., Bahgat W.M., Badawy M. (2021). A CNN based framework for classification of Alzheimer’s disease. Neural Comput. Appl..

[17] Shamshad F., Khan S., Zamir S.W., Khan M.H., Hayat M., Khan F.S., Fu H. (2023). Transformers in medical imaging: A survey. Med. Image Anal..

[18] Zhao H., Ou L., Zhang Z., Zhang L., Liu K., Kuang J. (2025). The value of deep learning-based X-ray techniques in detecting and classifying K-L grades of knee osteoarthritis: A systematic review and meta-analysis. Eur. Radiol..

[19] Sharma L. (2021). Osteoarthritis of the Knee. N. Engl. J. Med..

[20] Alvarez-Garcia O., Fisch K.M., Wineinger N.E., Akagi R., Saito M., Sasho T., Su A.I., Lotz M.K. (2016). Increased DNA Methylation and Reduced Expression of Transcription Factors in Human Osteoarthritis Cartilage. Arthritis Rheumatol..

[21] Liu Z., Lu T., Ma L., Zhang Y., Li D. (2024). DNA Demethylation of Promoter Region Orchestrates SPI-1-Induced ADAMTS-5 Expression in Articular Cartilage of Osteoarthritis Mice. J. Cell. Physiol..

[22] Papageorgiou A.-A., Litsaki M., Mourmoura E., Papathanasiou I., Tsezou A. (2023). DNA Methylation Regulates Sirtuin 1 Expression in Osteoarthritic Chondrocytes. Adv. Med. Sci..

[23] Zhu X., Chen F., Lu K., Wei A., Jiang Q., Cao W. (2019). PPAR*γ* Preservation via Promoter Demethylation Alleviates Osteoarthritis in Mice. Ann. Rheum. Dis..

[24] Pharo H., Vedeld H.M., Sjurgard I.V., Pinto R., Lind G.E. (2025). From concept to clinic: A roadmap for DNA methylation biomarkers in liquid biopsies. Oncogene.

[25] Arruda A.L., Katsoula G., Chen S., Reimann E., Kreitmaier P., Zeggini E. (2024). The Genetics and Functional Genomics of Osteoarthritis. Annu. Rev. Genom. Hum. Genet..

[26] Scerif F., Eldridge S.E. (2026). Osteoarthritis year in review 2025: Biology. Osteoarthr. Cartil..

[27] Hatzikotoulas K., Southam L., Stefansdottir L., Boer C.G., McDonald M.L., Pett J.P., Pedersen O.B. (2025). Translational genomics of osteoarthritis in 1,962,069 individuals. Nature.

[28] Zhou X., Ye X., Yao J., Lin X., Weng Y., Huang Y., Nong L. (2025). Identification and validation of transcriptome-wide association study-derived genes as potential druggable targets for osteoarthritis. Bone Jt. Res..

[29] Mei L., Zhang Z., Chen R., Liu Z., Ren X., Li Z. (2023). Identification of candidate genes and chemicals associated with osteoarthritis by transcriptome-wide association study and chemical-gene interaction analysis. Arthritis Res. Ther..

[30] Li T., Chubinskaya S., Esposito A., Jin X., Tagliafierro L., Loeser R., Spagnoli A. (2019). TGF-*β* type 2 receptor–mediated modulation of the IL-36 family can be therapeutically targeted in osteoarthritis. Sci. Transl. Med..

[31] Chen B., Liu X., Hu M., Liao J. (2025). Insights into the bone morphogenetic protein signaling in musculoskeletal disorders: Mechanisms and crosstalk. J. Orthop. Transl..

[32] Shen J., Li S., Chen D. (2014). TGF-*β* signaling and the development of osteoarthritis. Bone Res..

[33] Thielen N.G.M., van der Kraan P.M., van Caam A.P.M. (2019). TGF-*β*/BMP signaling pathway in cartilage homeostasis. Cells.

[34] Wu M., Chen G., Li Y.P. (2016). TGF-*β* and BMP signaling in osteoblast, skeletal development, and bone formation, homeostasis and disease. Bone Res..

[35] Cao X., Wu S., Wang X., Huang J., Zhang W., Liang C. (2023). Receptor tyrosine kinase C-kit promotes a destructive phenotype of FLS in osteoarthritis via intracellular EMT signaling. Mol. Med..

[36] Wu M., Wu S., Chen W., Li Y.P. (2024). The roles and regulatory mechanisms of TGF-*β* and BMP signaling in bone and cartilage development, homeostasis and disease. Cell Res..

[37] Ghafouri-Fard S., Poulet C., Malaise M., Abak A., Mahmud Hussen B., Taheriazam A., Hallajnejad M. (2021). The emerging role of non-coding RNAs in osteoarthritis. Front. Immunol..

[38] Gu J., Rao W., Huo S., Fan T., Qiu M., Zhu H., Sheng X. (2022). MicroRNAs and long non-coding RNAs in cartilage homeostasis and osteoarthritis. Front. Cell Dev. Biol..

[39] Sun K., Luo J., Guo J., Yao X., Jing X., Guo F. (2020). The PI3K/AKT/mTOR signaling pathway in osteoarthritis: A narrative review. Osteoarthr. Cartil..

[40] Yao Q., Wu X., Tao C., Gong W., Chen M., Qu M., Xiao G. (2023). Osteoarthritis: Pathogenic signaling pathways and therapeutic targets. Signal Transduct. Target. Ther..

[41] Liao J., Wu T., Zhang Q., Shen P., Huang Z., Wang J., Chen G. (2026). TGF-*β*/BMP signaling in skeletal biology: Molecular mechanisms, regulatory networks, and therapeutic implications in development, regeneration, and disease. Bone Res..

[42] Jurić I., Todorović P., Kelam N., Boban D., Bajt P., Racetin A., Vukojević K. (2025). MAPK Pathway Activation Patterns in the Synovium Reveal ERK1/2 and EGFR as Key Players in Osteoarthritis. Biomedicines.

[43] Sheng W., Wang Q., Qin H., Cao S., Wei Y., Weng J., Zeng H. (2023). Osteoarthritis: Role of peroxisome proliferator-activated receptors. Int. J. Mol. Sci..

[44] Morikawa M., Derynck R., Miyazono K. (2016). TGF-*β* and the TGF-*β* family: Context-dependent roles in cell and tissue physiology. Cold Spring Harb. Perspect. Biol..

[45] Sun Y., Cai H., Ge J., Shao F., Huang Z., Ding Z., Zang Y. (2022). Tubule-derived INHBB promotes interstitial fibroblast activation and renal fibrosis. J. Pathol..

[46] Wang H., Yuan T., Wang Y., Liu C., Li D., Li Z., Sun S. (2024). Osteoclasts and osteoarthritis: Novel intervention targets and therapeutic potentials during aging. Aging Cell.

[47] He Y., Zhu W., Alexander P.G., Hines S.E., Bartholomew O.G., Zhao C., Lin H. (2025). Forkhead box O proteins in chondrocyte aging and diseases. J. Orthop. Transl..

[48] Han Z., Wang K., Ding S., Zhang M. (2024). Cross-talk of inflammation and cellular senescence: A new insight into the occurrence and progression of osteoarthritis. Bone Res..

[49] Adam R.C., Pryce D.S., Lee J.S., Zhao Y., Mintah I.J., Min S., Gusarova V. (2023). Activin E–ACVR1C cross talk controls energy storage via suppression of adipose lipolysis in mice. Proc. Natl. Acad. Sci. USA.

[50] Park S.Y., Cho Y., Son S.M., Hur J.H., Kim Y., Oh H., Choi C.S. (2025). Activin E is a new guardian protecting against hepatic steatosis via inhibiting lipolysis in white adipose tissue. Exp. Mol. Med..

[51] Cabot J.H., Ross E.G. (2023). Evaluating prediction model performance. Surgery.

[52] Lü L., Chen D., Ren X.L., Zhang Q.M., Zhang Y.C., Zhou T. (2016). Vital nodes identification in complex networks. Phys. Rep..

